# Strain Transfer Characteristics of Multi-Layer Optical Fiber Sensors with Temperature-Dependent Properties at Low Temperature

**DOI:** 10.3390/s21020495

**Published:** 2021-01-12

**Authors:** Taolue Yang, Huaping Wang, Xingzhe Wang

**Affiliations:** Key Laboratory of Mechanics on Western Disaster and Environment, MoE, Key Laboratory of Special Function Materials and Structure Design, College of Civil Engineering and Mechanics, Lanzhou University, Lanzhou 730000, China; yangtl16@lzu.edu.cn (T.Y.); hpwang@lzu.edu.cn (H.W.)

**Keywords:** optical fiber sensor, strain transfer analysis, strain measurement, low temperature, non-uniform temperature distribution

## Abstract

Optical fiber sensors have been potentially expected to apply in the extreme environment for their advantages of measurement in a large temperature range. The packaging measure which makes the strain sensing fiber survive in these harsh conditions will commonly introduce inevitable strain transfer errors. In this paper, the strain transfer characteristics of a multi-layer optical fiber sensing structure working at cryogenic environment with temperature gradients have been investigated theoretically. A generalized three-layer shear lag model incorporating with temperature-dependent properties of layers was developed. The strain transfer relationship between the optical fiber core and the matrix has been derived in form of a second-order ordinary differential equation (ODE) with variable coefficients, where the Young’s modulus and the coefficients of thermal expansion (CTE) are considered as functions of temperature. The strain transfer characteristics of the optical sensing structure were captured by solving the ODE boundary problems for cryogenic temperature loads. Case studies of the cooling process from room temperature to some certain low temperatures and gradient temperature loads for different low-temperature zones were addressed. The results showed that different temperature load configurations cause different strain transfer error features which can be described by the proposed model. The protective layer always plays a main role, and the optimization geometrical parameters should be carefully designed. To verify the theoretical predictions, an experiment study on the thermal strain measurement of an aluminum bar with optical fiber sensors was conducted. LUNA ODiSI 6100 integrator was used to measure the Rayleigh backscattering spectra shift of the optical fiber at a uniform temperature and a gradient temperature under liquid nitrogen temperature zone, and a reasonable agreement with the theory was presented.

## 1. Introduction

Optical fiber sensors, possessing great advantages of high sensitivity and flexibility, immunity to electromagnetic interference, light weight and small size, and the ability to provide multiplexed or distributed sensing, are extensively attracted in various engineering applications. As one of important kind of sensor technologies, fiber grating sensors have been widely studied and commercialized for health monitoring and oil industries, which are used to measure strain, temperature, pressure, and other quantities by modifying a fiber so that the quantity to be measured modulates the intensity, phase, polarization, wavelength or transit time of light in the fiber [1,2,3,4]. Meanwhile, other rather mature optical fiber sensor technologies such as optical time-domain reflectometers, optical frequency-domain reflectometers (OFDRs), fiber-optic gyroscopes, and optical fiber current sensors also have been attracted broad attentions [5]. 

So far, various optical fiber sensors have become more powerful tools in the traditional fields, for example bridges, high-speed railways, aircrafts, etc. [6,7,8,9]. However, the harsh environments such as extreme low to high temperatures, shock, radiation, corrosive conditions, high radio-frequency interference and pressure, give rise to unique challenges and opportunities to fiber optic sensors. There were some efforts in developing optical fiber sensors for harsh environments due to the excellent properties of silica fiber. By the way of the Bragg grating written into silica with femtosecond lasers using either the phase mask method or the point-by-point method, fiber Bragg gratings (FBGs) could be used for sensing strain and/or temperature in environment at temperatures less than 1000 °C [10]. Consider the most widely used optical fiber material, fused silica, being incapable of withstanding the chemically corrosive environments, the sapphire-FBG based temperature sensor was fabricated and packaged to show great linearity of temperature response from room temperature to elevated temperature [11]. For structural health monitoring of the next-generation of nuclear reactors, different technologies for realizing temperature resistant FBGs were developed for temperature and strain measurements especially for components exposed to high temperature and radiation levels [12]. 

On the other hand, some particular interests are extreme low temperature as low as a few Kelvin environment, for example, helium or hydrogen gas leak detection in cryogenic condition is critically important in the production and use of liquid fuels. Other applications in aerospace vehicles, superconducting magnets, and high-energy physics experiments also involve advanced technologies and devices designed to operate in cryogenic environments [13,14,15,16,17]. In 2019, the National High Magnetic Field Laboratory of USA used hybrid superconducting magnets to achieve the highest magnetic field of 45.5T to date [18]. The current carried in superconducting magnets can reach thousands of amps, where if the huge electromagnetic energy is improperly controlled it will result in a disaster and quench for the high-field magnets. At present, the quenching mechanism of superconducting magnets is not completely clear, and there is some contingency. Plenty of studies have found that the uncertainty of the quenching in superconducting magnets is largely due to forces during their operation. The method based on internal strain measurement of the magnet can detect the abnormal temperature and electromagnetic force before the expansion of the quench hot spot and start the safety measures in time [19]. Fiber optic sensor will be the best choice in this area, because it can effectively monitor the temperature and strain inside the magnet with embedded technology, and has great advantages such as electromagnetic immunity, small size, corrosion resistance and low loss. 

Low temperature environments also pose a challenge to existing sensing technologies. The performances of conventional room-temperature sensors, including sensitivities, response times, and lifetime, degrade rapidly when temperature gets lower. A few applications using optical fiber sensors at cryogenic temperatures have been developed lately, such as FBGs embedded in or bonded to substrates (e.g., PMMA, Teflon) with larger thermal expansion coefficients for overcoming their low temperature sensitivity, a continuous liquid level sensing system for liquid nitrogen and helium tanks [11,17].And some optical fiber sensors including FBG, Raman-scattering, Rayleigh-scattering and Brillouin-scattering for monitoring cryogenic temperature of high-temperature superconducting tapes at 77 K or even lower temperature have been attempted [20,21,22,23]. In these investigations, optical fiber sensors were mainly developed to measure cryogenic temperature in which the deformation of the materials and structures commonly were not considered.

The concept of strain transfer is originated from the deformation transfer between fiber-reinforced composite matrix and fibers. It has been adopted to describe the transfer relationship between the sensing fiber and the test object with the development of optical fiber sensors. Since the 1990s, researchers have used elastic mechanics in cylindrical coordinates for modeling their behaviors [24]. Subsequent improvements have been made, and the shear lag model of micromechanics of composite materials has been put forward. Ansari and Yuan [25] firstly proposed the shear lag model of a three-layer structure including a fiber core, a protective layer and a matrix, and LeBlanc [26] further merged the protective layer with the matrix and gave a shear lag model of a two-layer structure. Subsequently, Li et al. [27,28] studied the case that the mechanical model of strain transfer under non-axial forces, in which two layers of the three-layer structure were sheared. Feng et al. [29] gave the strain transfer relationship in a four-layer structure with cracks. Sun et al. [30] focused on a desensitization method to develop a wide-range FBG sensor for extra-large strain monitoring and improve the accuracy. Wang et al. [31] reviewed the development of several classic strain transfer theories and used Goodman’s hypothesis to obtain a model for asphalt pavements. Recently, the homemade polymer-FBG sensors embedded in coils were used to measure the strain responses during excitation and quench training tests. Compared to the cryogenic resistance strain gauges with complex compensation bridges, the polymer-FBG sensors exhibit more advantages to record the internal strain in the magnets [32]. However, the sensors were operated at extreme cryogenic environments around 5 K so that the thermal sensitivity of FBG can be disregarded and only the strains were measured. 

Although a few attempts of optical fiber sensors to low temperature usage were carried out, challenges still exist in extreme environments. The key part of optical fibers is a thin fiber core made of glass covered with a polymer layer to achieve toughening. To make the sensing fiber survive in harsh conditions, additional packaging measures are required, which not only plays a protective role, but also realizes a variety of sensing functions by using different structures and functional materials. The polymer material as the adhesive and protective layer expands and softens at high temperatures, and shrinks and cracks at low temperatures, which causes measurement errors. For strain sensing, it is the first requirement to truly reflect the strain information of the structure in tests. Because the protective layer isolates the sensing optical fiber core from the test object, a deformation difference occurs which is defined as strain transfer error. Strain transfer theory describing the transfer relationship between the sensing fiber and the test object is proposed to correct this error and to improve the measurement accuracy [25,26,27,28,29]. It can be found that the composite structures of optical fiber sensors in the abovementioned studies are mostly subjected to uniform deformations at normal temperature. However, most optical fiber sensors are sensitive to temperature and strain, which always are mixed. In extreme low temperature environments, the heat can be completely transferred to the different layers of the sensors, and materials properties like Young’s modulus, coefficients of thermal expansion of those layers are always temperature dependence. Additionally, in the distributed optical fiber sensor, the micro sensor in length of an order of millimeter is evenly distributed on the optical fiber with a density of several hundred per meter. In the case of large strain or temperature gradient, for example the temperature gradient of a high-temperature superconductor structure up to 65 K/cm during a quench process [22], the distributed optical fiber sensor can measure the strain distribution properly. Strain transfer errors dependent on the load configuration will vary greatly.

This study aims to analyze the strain transfer characteristics of distributed optical fiber sensors at low temperature under non-uniform loads. It is of important engineering significance to study whether the response of each position of distributed fiber reflects the true value under the extreme conditions of cryogenic temperature and large gradient. In consideration of the temperature-dependent material properties, a generalized three-layer (e.g., fiber core, protective layer and matrix) shear lag model incorporated with temperature-dependent properties for describing the strain transfer relationship between the matrix and the fiber core was developed. To examine the strain transfer response of optical fiber sensor at low-temperature zone, several temperature loads, such as uniform temperature, linearly gradient and Gaussian distributed temperature, were addressed. The correlated sensitive parameters of the sensing model on the strain transfer ratio were discussed detailly. Additionally, an experiment study was conducted to verify the theoretical predictions and the Rayleigh backscattering spectra shifts associated with thermal strain measurement of aluminum bars with optical fiber embedded were measured.

## 2. Multi-Layer Strain Transfer Model and Analysis

### 2.1. Fundamental Equations

**Figure 1:**
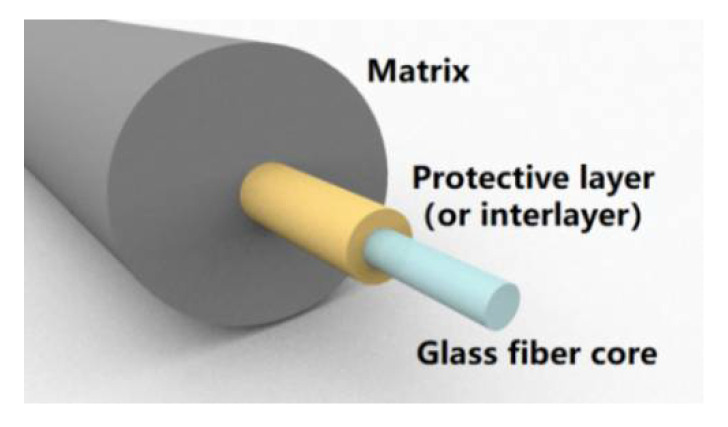
Structure of embedded optical fiber sensing model.

An ideal embedded optical fiber sensing model can be briefly described as shown in Figure 1, which is a typical sensor structure commonly used for sensing strain and/or temperature. The core sensing element of the optical fiber is glass fiber core, which is coated with a thin polymer interlayer and a thick matrix layer. The Young’s modulus of the thin interlayer is much lower than that of the fiber core or matrix, which is unfavorable for strain transfer. The lower Young’s modulus means greater deformation during stretching, shearing and torsion. The interlayer will absorb a part of the strain from the matrix layer. The strain of the matrix can be caused by external mechanical and temperature loads. The glass fiber core does not directly sense the mechanical load, but indirectly feel the strain through the interlayer. Different from mechanical load, temperature load will directly affect layers simultaneously. 

Generally, in order to meet various working conditions, the interlayer is usually a multi-layer structure. Considering it as a single equivalent layer will greatly reduce the difficulty of theoretical analysis. Additionally, several assumptions are adopted to simplify the theoretical modeling for establishing a relatively concise equation, such as the interfaces of the three-layer structure are perfectly combined without interfacial slip, only normal stress in the fiber core due to the small radius is considered. 

Since the sensing model and the operation environment, the matrix of the optical fiber sensor is assumed only undergoing normal strain in the axial direction. Further, the strain response of the matrix to the glass fiber core is transmitted through the protective interlayer. The deformation in the protective interlayer generates a buffering effect in the mechanical model, so that its mechanical properties are critically important for such sensing structure. 

**Figure 2:**
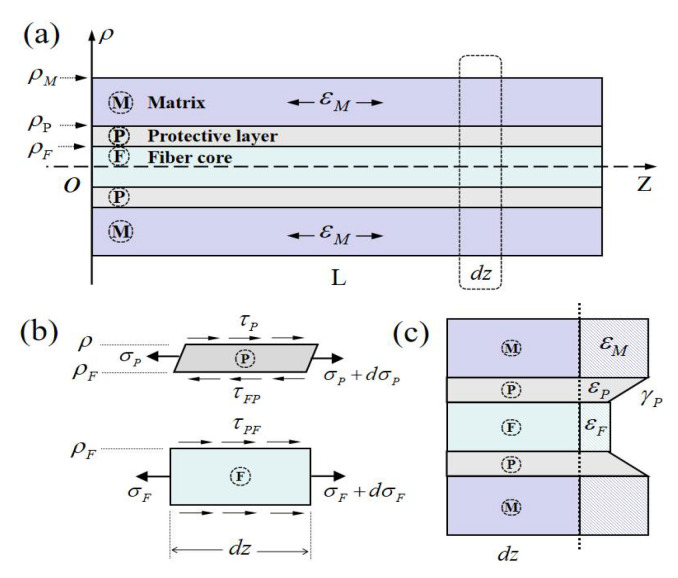
Sketch of multi-layer structure of optical fiber sensor: (**a**) longitudinal section; (**b**) stress states for the protective layer and fiber core; (**c**) deformation profile of multiple layers.

The structure and stress state of the three-layer optical fiber sensor is shown in Figure 2a. The optical fiber length is L, the radial coordinate denotes as ρ, and the central axis denotes as z. The three layers of the structure are respectively denoted by the subscripts of *F*, *P* and *M*, corresponding to the fiber core, protective layer and matrix layer. Figure 2b,c illustrate the stresses and deformation of infinitesimal elements of different layers. Because of the extreme environment of the optical fiber sensors, for example, the extra-low temperature, the material properties are always the temperature-dependent. The Young’s modulus and coefficients of thermal expansion of the protective layer and matrix are considered as functions of temperature, $E_{i}=E_{i}(T)$, $\alpha_{i}=\alpha_{i}(T)$, (i=F,P,M). Furthermore, for a measurement of a structure in temperature gradient environment or a long distribution continuous fiber with multiple sensors, the temperature along the fiber can be not be neglected so that T=T(z). In such cases, the material properties will be function of *z*-axis.

The equilibrium equation for the glass fiber core along z-axial, from Figure 2b, can be obtained in the form [24],

(1) $$\frac{d\sigma_{F}}{dz}+\frac{2\tau_{PF}}{\rho_{F}}=0$$

where $\sigma_{F}$, $\tau_{PF}$ are the axial stress (the normal stress parallel to the *z*-axis) and shear stress (parallel to the *z*-axis on the cylindrical surface of the columnar micro-element body), $\rho_{F}$ denotes the radius of the fiber core. 

For the protective layer, the equilibrium equation is given as [28],

(2) $$\frac{\rho^{2}-\rho_{F}^{2}}{2\rho }\frac{d\sigma_{P}}{dz}-\frac{\rho_{F}}{\rho }\tau_{FP}+\tau_{P}(\rho ,z)=0$$

where $\sigma_{P}$ is the axial stress in the protective layer, $\tau_{P}$ represents the shear stress at ρ along the radial direction, $\tau_{PF}$ is the shear stress at the interface between the fiber core and protective layer. Because of the ideal interface between two materials, the continuity conditions of interface stresses exist, $\tau_{PF}=\tau_{FP}$.

From Equations (1) and (2), one can get the shear stress of the protective layer in the following form,

(3) $$\tau_{P}(\rho ,z)=\frac{\rho_{F}^{2}}{2\rho }\frac{d}{dz}(\sigma_{P}-\sigma_{F})-\frac{\rho }{2}\frac{d\sigma_{P}}{dz}$$

The relative displacement between the matrix and fiber core can be obtained by shear deformation of the protective layer, which gives

(4) $$\begin{array}{c} u_{M}-u_{F}=\int_{\rho_{F}}^{\rho_{P}}\gamma_{P}(\rho ,z)d\rho =\frac{2(1+\mu_{P})}{E_{P}}\int_{\rho_{F}}^{\rho_{P}}\tau_{P}(\rho ,z)d\rho \\ =\frac{1+\mu_{P}}{E_{P}}\int_{\rho_{F}}^{\rho_{P}}\left[\frac{\rho_{F}^{2}}{\rho }\frac{d}{dz}(\sigma_{P}-\sigma_{F})-\rho \frac{d\sigma_{P}}{dz}\right]d\rho \end{array}$$

where the elastic shear constitutive relationship is used. $u_{M},u_{F}$ refer to the displacements of the matrix and fiber core along z-direction, respectively. And $E_{P},\mu_{P}$ are respectively the Young’s modulus and Poisson’s ratio of the protective layer. $\gamma_{P}(\rho ,z)$ is the shear strain of the protective layer at position (ρ,z).

To further simplify the above equation, the stresses in the glass fiber core and the protective layer can be expressed by the thermoelastic constitutive relationship

(5) $$\sigma_{F}=E_{F}\varepsilon_{Fe}=E_{F}(\varepsilon_{F}-\varepsilon_{FT}),\;\varepsilon_{FT}=\int_{T_{0}}^{T_{1}}\alpha_{F}dT$$

(6) $$\sigma_{P}=E_{P}\varepsilon_{Pe}=E_{P}(\varepsilon_{P}-\varepsilon_{PT}),\;\varepsilon_{PT}=\int_{T_{0}}^{T_{1}}\alpha_{P}dT$$

in which $\varepsilon_{Fe},\varepsilon_{Pe}$ and $\varepsilon_{F},\varepsilon_{P}$ are respectively the elastic strains and total strains in the two materials, $\varepsilon_{FT},\varepsilon_{PT}$ denote the thermal strain caused by the temperature from $T_{0}$ to $T_{1}$, respectively. 

Taking differential operation on both sides of Equation (4) with respect to z, one can gain

(7) $$\begin{array}{c} \varepsilon_{M}-\varepsilon_{F}=(1+\mu_{P})\frac{d}{dz}\int_{\rho_{F}}^{\rho_{P}}\{\frac{\rho_{F}^{2}}{E_{P}\rho }\frac{d}{dz}[E_{P}(\varepsilon_{P}-\varepsilon_{PT})-E_{F}(\varepsilon_{F}-\varepsilon_{FT})]\\ -\frac{\rho }{E_{P}}\frac{d}{dz}[E_{P}(\varepsilon_{P}-\varepsilon_{PT})]\}d\rho \end{array}$$

Since the layers are very thin and the elastic parameters and strains are reasonably assumed to be independent with the radial coordinate. Additionally, due to the fiber core being strained together with the middle layer, the elastic strain gradients are expected to be of the same order [27], that is, $d\varepsilon_{Fe}/dz≅d\varepsilon_{Pe}/dz$.

By ignoring the high order infinitesimals related to modulus, Equation (7) further can be reduced into

(8) $$\begin{array}{cc} \varepsilon_{M}-\varepsilon_{F}=(1+\mu_{P})\rho_{F}^{2} & \{\ln\frac{\rho_{P}}{\rho_{F}}\frac{d}{dz}\{\frac{1}{E_{P}}\frac{d}{dz}[E_{P}(1-\frac{E_{F}}{E_{P}})(\varepsilon_{F}-\varepsilon_{FT})]\}\\ & +\frac{1}{2}(1-\frac{\rho_{P}^{2}}{\rho_{F}^{2}})\frac{d}{dz}\{\frac{1}{E_{P}}\frac{d}{dz}[E_{P}(\varepsilon_{F}-\varepsilon_{FT})]\}\} \end{array}$$

For the fiber core, it is a kind of silicas whose temperature-dependent effect can be omitted compared to that of the protective layer and matrix, i.e., $\varepsilon_{FT}\ll \varepsilon_{F},\varepsilon_{M}$. And the Poisson’s ratio and coefficient of thermal expansion of the fiber core are almost independent with the temperature, so that $d\alpha_{F}/dT≅0$. 

Therefore, Equation (8) is rewritten as

(9) $$\frac{d}{dz}[\frac{1}{E_{P}}\frac{d}{dz}(R_{1}E_{P}\varepsilon_{F})]+\varepsilon_{F}=\varepsilon_{M}$$

in which

(10) $$R_{1}=(1+\mu_{P})\rho_{F}^{2}[\frac{1}{2}(1-\frac{\rho_{P}^{2}}{\rho_{F}^{2}})+(1-\frac{E_{F}}{E_{p}})\ln\frac{\rho_{P}}{\rho_{F}}]$$

The above Equation (9) presents the relationship between strain of the fiber core and that of matrix material, and an important index for evaluating the performance of optical fiber sensing structures is called the strain transfer ratio, which is defined as $\eta =\varepsilon_{F}/\varepsilon_{M}$.

### 2.2. Nondimensional Forms of Equations

To get the general equations for the strain transfer characteristics for the multi-layer fiber sensor, the following nondimensional valuables are introduced,

(11) $$\bar{z}=\frac{z}{L},\;\bar{L}=\frac{L}{\rho_{F}},\;\bar{\rho }=\frac{\rho_{P}}{\rho_{F}},\;{\bar{E}}_{P}=\frac{E_{P}}{E_{F}}$$

Then, Equation (9) can be rewritten in a nondimensional form

(12) $$\frac{d}{d\bar{z}}[\frac{1}{{\bar{E}}_{P}}\frac{d}{d\bar{z}}({\bar{R}}_{1}{\bar{E}}_{P}\varepsilon_{F})]+\varepsilon_{F}=\varepsilon_{M}$$

in which

(13) $${\bar{R}}_{1}=\frac{1+\mu_{P}}{{\bar{L}}^{2}}[\frac{1}{2}(1-{\bar{\rho }}^{2})+(1-\frac{1}{{\bar{E}}_{P}})\ln\bar{\rho }]$$

We further can get the general form of Equation (12) as follows,

(14) $${\bar{R}}_{1}\frac{d^{2}\varepsilon_{F}}{d{\bar{z}}^{2}}+{\bar{R}}_{2}\frac{d\varepsilon_{F}}{d\bar{z}}+{\bar{R}}_{3}\varepsilon_{F}=\varepsilon_{M}$$

in which

(15) $${\bar{R}}_{2}=2\frac{d{\bar{R}}_{1}}{d\bar{z}}+\frac{{\bar{R}}_{1}}{{\bar{E}}_{P}}\frac{d{\bar{E}}_{P}}{d\bar{z}},\;{\bar{R}}_{3}=\frac{d}{d\bar{z}}(\frac{d{\bar{R}}_{1}}{d\bar{z}}+\frac{{\bar{R}}_{1}}{{\bar{E}}_{P}}\frac{d{\bar{E}}_{P}}{d\bar{z}})+1$$

It can be found that Equation (14) is a second-order ordinary differential equation with variable coefficients, which is commonly difficult to solve with an analytical method. However, when we consider the general usage of the optical fiber sensor in a conventional manner, for example, the material properties are independent with temperature and one can easily to get ${\bar{R}}_{2}=0$, ${\bar{R}}_{3}=1$. Equation (14) reduces into a simple one

(16) $$\frac{d^{2}\varepsilon_{F}}{d{\bar{z}}^{2}}+\frac{1}{{\bar{R}}_{1}}\varepsilon_{F}=\frac{1}{{\bar{R}}_{1}}\varepsilon_{M}$$

which is the same as that in the literature for a three-layer fiber sensor developed by Li et al. [27,28]. It indicated that the present generalized model can be degenerated into a simple one as reported in the literatures.

Additionally, the fiber sensor is assumed to be free from axial stress at both ends due to the matrix material being non-contact with the fiber beyond the ends of the interface between the fiber core and the protective layer. It makes to use a boundary condition of zero of the strain transferred to the optical fiber core at both ends of the fiber,

(17) $${\varepsilon_{F}|}_{\bar{z}=0}=0,\;{\varepsilon_{F}|}_{\bar{z}=1}=0$$

### 2.3. Numerical Solution to the ODE

For the second-order ODE with variable coefficients (e.g., Equations (14) or (16)) and boundary conditions (Equation (17)), the shooting method is utilized for the numerical solution. The well-developed method takes its name from the situation in the two-point boundary value problem for a second-order differential equation with initial and final values of the solution prescribed. The two-point boundary value problem is treated as an initial value problem, in which $\bar{z}$ plays the role of the time variable, with $\bar{z}=0$ being the “initial time” and $\bar{z}=1$ being the “final time”. Varying the initial slope gives rise to a set of profiles which suggest the trajectory of a projectile “shot” from the initial point. 

To obtain high-precision numerical solution of the differential equation, the explicit fourth order Runge-Kutta approach was used. When we consider the simplified case of conventional manner where material properties of multi-layer are independent with temperature. The solution of Equation (16) can be fortunately obtained in an analytical form,

(18) $$\varepsilon_{F}=C_{1}\sinh(\sqrt{-1/{\bar{R}}_{1}}\bar{z})+C_{2}\cosh(\sqrt{-1/{\bar{R}}_{1}}\bar{z})+\varepsilon_{M}$$

Here, the constants of integration, $C_{1},C_{2}$ can be determined easily from the boundary conditions of Equation (17).

## 3. Results and Discussions

### 3.1. Temperature-Dependent Material Properties

For most of the optical fiber sensors, the materials properties of their composite structures are temperature dependent, especially in a large range of temperature zone. As the temperature gradually drops, material properties of the sensing structure show considerable differences compared to that at room temperature.

Generally, the temperature dependence of material properties, for example Young’s modulus and the coefficients of thermal expansion, can be expressed in the form of polynomial as follow [32,33,34],

(19) $$E_{i}\left(T\right)=a_{i0}+a_{i1}T+a_{i2}T^{2}+a_{i3}T^{3}+a_{i4}T^{4}+a_{i5}T^{5}$$

(20) $$\alpha_{i}\left(T\right)=b_{i0}+b_{i1}T+b_{i2}T^{2}+b_{i3}T^{3}+b_{i4}T^{4}$$

where the subscripts i(=F,P,M) represent the different layers of the sensor structure, $a_{ij}(j=0,1,\cdots ,5)$ and $b_{ij}(j=0,1,\cdots ,4)$ are the fitting coefficients from experiments. 

**Figure 3:**
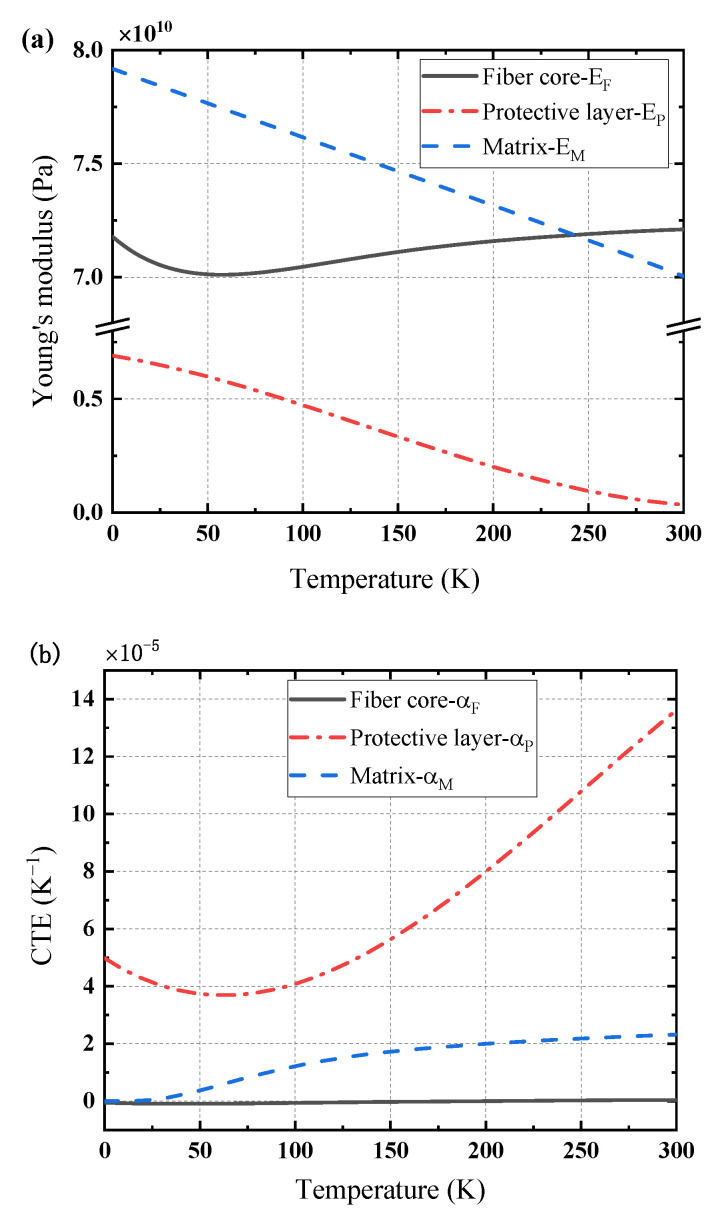
Material properties of multiple layers dependent with temperature: (**a**) Young’s modulus, (**b**) CTE.

One possible and common material combination of the matrix, the protective layer, and the fiber core can be aluminum, Teflon, and glass, respectively. Figure 3 illustrates their Young’s modulus and the coefficients of thermal expansion dependent upon temperature in a large zone [33,34]. One can see that, compared to those of glass fiber core, the properties of matrix and protective layer show significant variations with temperature. For Young’s modulus, the values of the matrix and protective layer increase linearly as temperature drops. While the coefficients of thermal expansion show quiet noteworthy feature for the matrix and protective layer, the value of matrix slightly decreases and that of the protective layer firstly drops and reaches a minimum at about 60 K then increases with temperature decreasing. For different materials, their properties have great differences which may not omitted in a large temperature zone or temperature gradient environment as they work.

### 3.2. Different Temperature Loads

For a structure under mechanical loads, there are abundant investigations on its deformation measured with optical fiber sensors, to show the effectiveness at work. The strain-temperature simultaneous sensing characteristics also have been considered, where the interference caused by temperature when measuring strain can usually be solved by temperature compensation technology, such as temperature compensation block, thermometer, dual optical fiber, etc. Here, we will mainly consider the temperature loads. As a structure experienced to temperature loads, there are usually two thermal equilibrium states. One situation is that the temperature of the whole structure is spatially uniform so that the temperature gradient can be ignored. The other is that temperature in the structure is temporally constant and a temperature gradient distribution may exist. Especially, in structures with a heat source, the temperature distribution is similar to the Gaussian bell curve, and the temperature gradient changes sharply, with obvious peaks. In the following examples, we will consider these two cases.

#### 3.2.1. Uniform Temperature Load

Consider a cooling process, the thermal deformation will occur inside the structure due to expansion and contraction of materials. The multi-layer sensing models made of different materials can also cause local stress due to thermal mismatch. Since the CTE and the Young’s modulus show a non-linear relationship with temperature, the internal stress and the amplitude of temperature change also show a non-linear relationship. However, for a simple case of the uniform temperature load, the material properties at a temperature can be determined from the temperature-dependent curve of experiments. 

The cooling process from room temperature (e.g., $T_{0}=293\;K$) to some certain low temperature $T_{1}$ is concerned. The strain in the matrix is easily evaluated by $\varepsilon_{M}=\int_{T_{0}}^{T_{1}}\alpha_{M}dT$. The thermally deformed matrix transfers its strain to the optical fiber core through the protective layer, and the strain measured in the fiber sensor can be obtained from the theoretical model. 

**Figure 4:**
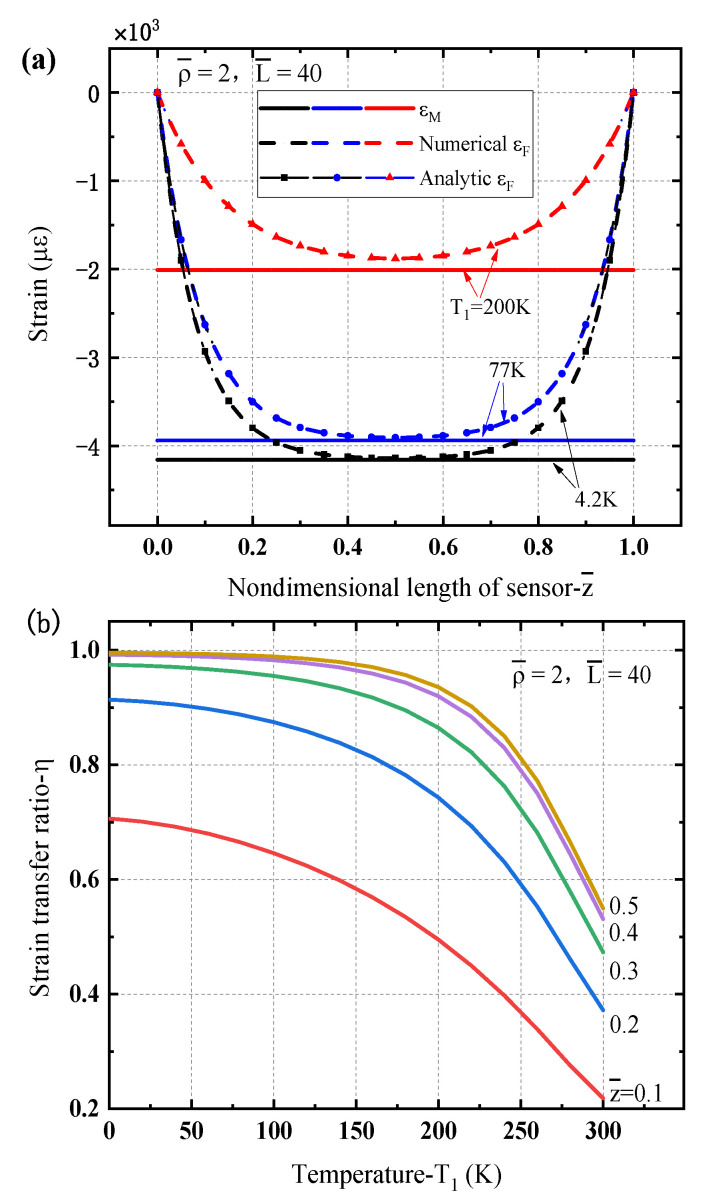
Strain transfer characteristic of the optical fiber sensor under a uniform temperature drop: (**a**) strains in matrix and fiber sensor for different temperature; (**b**) strain transfer ratio dependence of temperature at different locations along the fiber sensor.

Figure 4 shows the strain transfer characteristic of the optical fiber sensor under a uniform temperature drop. Since the fiber material has small CTE, the thermal deformation of the glass fiber core is far less than that of the matrix. The matrix will pull the fiber core through the protective layer, so that the fiber core and the matrix shrink simultaneously. One can see that, from Figure 4a, for different temperature drops (e.g., $T_{1}=200\;K,77\;K,4.2\;K$) the matrix is in constriction with different constant stains while the strain in the fiber core also is compressed with a U-shape. At the end positions of the optical fiber sensor (e.g., $\bar{z}=0,1$), the strains are zeroes, while the strain at the midpoint of the fiber core is close to that of the matrix. The analytical results also present a good agreement with the numerical predictions, as shown in Figure 4a. For a lower temperature (e.g., 4.2 K or 77 K) there has a quite large region where the strain in the fiber core is much closer the strain in the matrix. It means the measured strain by the sensor is consistent with the real strain of the matrix and a good performance of strain transfer is obtained. In other words, the optical fiber sensor exhibits a good capability and effectiveness at low temperatures. The reason is mainly because that the young’s modulus of the protective layer increases with the decrease of temperature to increase a good performance of the strain transfer ratio. Figure 4b further illustrates the strain transfer ratio of the optical fiber sensor dependence of the temperature decreases at different locations. It clearly shows that the strain transfer ratio decreases with temperature $T_{1}$, especially for a high temperature, and the value at the midpoint (i.e., $\bar{z}=0.5$) is almost 1.0 in a quite large range of low temperature which is larger than other locations along the sensor length.

**Figure 5:**
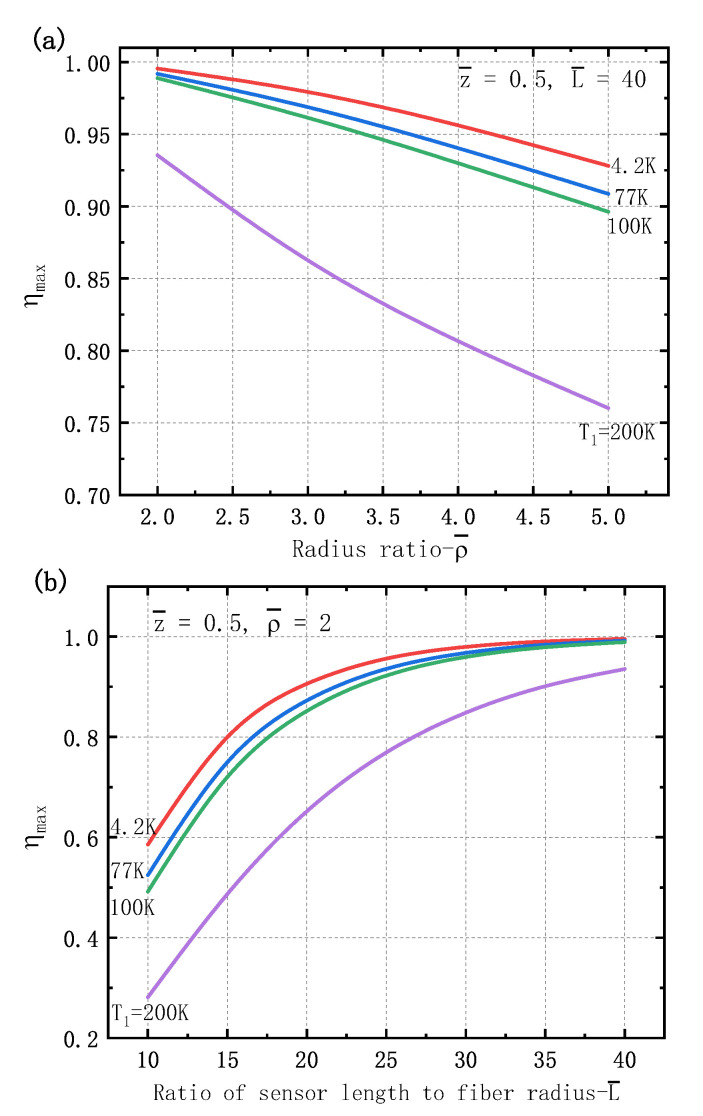
The maximum strain transfer ratio dependence of geometrical parameters of sensor under a uniform temperature drop: (**a**) radius ratio $\bar{\rho }$; (**b**) ratio of sensor length to fiber radius $\bar{L}$.

The performance of the optical fiber sensor is usually relied on their geometrical parameters. Figure 5 illustrates the maximum strain transfer ratio of the fiber sensor for different temperate drops. Figure 5a plots the strain transfer ratio of the sensor dependence on the relative radius ratio of the protective layer, to shows the decrease as the radius ratio $\bar{\rho }$ increases. It is because the increase of the radius of the protective layer leads to transferred deformation from the matrix to the fiber core being much smaller. Figure 5b shows the maximum strain transfer ratio greatly increases as the fiber sensor length. When the ratio of sensor length to fiber radius $\bar{L}$ is larger than 30, the maximum strain transfer ratio is up to about 1.0 for cooling temperature $T_{1}$ less than 100 K. While for a small temperature drop, for example $T_{1}=200\;K$, the strain transfer ratio is a little small and it increase with the sensor length. The longer sensor length means that the accumulated drag effect of the interface induces more deformation in the fiber core. One can see that at a low temperature, thinner protective layer and longer embedded sensor length can bring about a large strain transfer ratio, which is consistent with that in the literature for a three-layer fiber sensor [28].

#### 3.2.2. Gradient Temperature Load

Non-uniform temperature loads subjected to structures are common in practice. Structures in the cooling or heating process usually involve a large temperature gradient, for instance, the temperature gradient in a superconductor structure up to 65 K/cm during a quench process [22], and a high gradient about 200–300 K/cm for a laser heating on an absorbing layer [35]. Therefore, for a gradient temperature load on a structure, at different positions the temperature is not consistent anymore, and the material properties will be spatial dependence. The equation used to describe the microelements changes with the position. The thermoelastic response of the fiber core to the matrix will be different from that of the uniform temperature load. In this section, we will consider two cases for the optical fiber sensing structure affected by gradient temperature loads.

(a) Linear Temperature Distribution Load

In this case, we consider that a linear gradient temperature distributes along the sensing structure in the form of $T_{1}(\bar{z})=T_{0}+\bar{z}\cdot \Delta T$, where the thermal deformation reference temperature denotes as $T_{0}$ (e.g., $T_{0}=4.2\;K,77\;K$) and ΔT means the temperature increases (e.g., ΔT=30 K,50 K,70 K). The thermoelastic strain in the matrix is evaluated by $\varepsilon_{M}\left(\bar{z}\right)=\int_{T_{0}}^{T_{1}(\bar{z})}\alpha_{M}(T)dT$. Additionally, since the strain transfer ratio η is different along the sensor, an average strain transfer ratio is introduced for the sensing structure with $\bar{\eta }=\int_{0}^{1}\eta d\bar{z}$.

**Figure 6:**
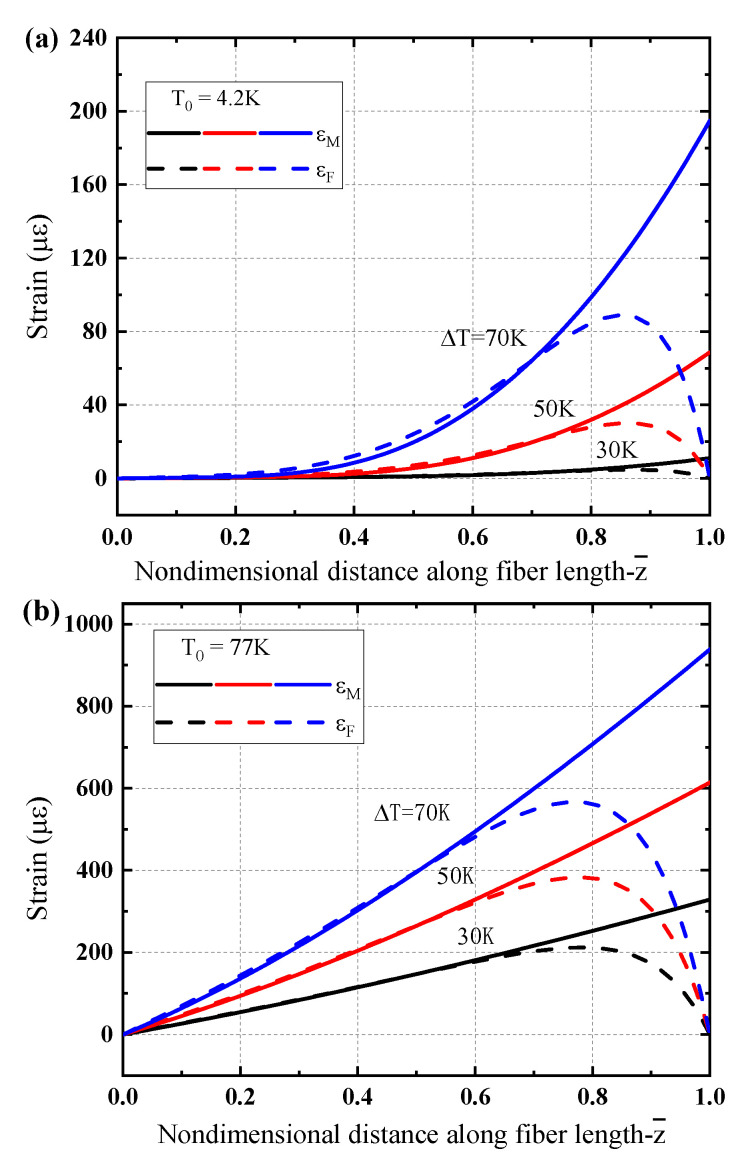
Strain characteristic of the optical fiber sensor under a linear gradient temperature load: (**a**) $T_{0}=4.2\;K$, (**b**) $T_{0}=77\;K$.

Figure 6 presents the strain distributions in the matrix and the fiber core of the sensing structure under a linear gradient temperature respectively for different low temperature regions. Figure 6a,b respectively show the results for $T_{0}=4.2\;K$ and $T_{0}=77\;K$. One can see that the thermal strain in the matrix does not show a linear increase as the temperature gradient distribution, but exhibits a concave form for liquid helium temperature region ($T_{0}=4.2\;K$) as shown in Figure 6a. While the strain shows almost linear increase along the sensor length for liquid nitrogen temperature zone ($T_{0}=77\;K$) as plotted in Figure 6b. The feature becomes more obvious as the gradient value ΔT is high, which is because the coefficient of thermal expansion of the matrix increases with the increase of temperature, and this trend is more prominent at $T_{0}=4.2\;K$ than that at $T_{0}=77\;K$. Although there is lack of the experiment observations for direct comparison, the thermoelastic strain induced by linear gradient temperature considered here is in analogy with the almost linear gradient bending strain in a small region measured for a cantilever [36]. Additionally, due to boundary conditions of sensing structure, the deformation of the fiber core is forced to zero at both ends. Near the left end, the strain consistency in the matrix and fiber core presents better situation, while near the right end a more obvious difference between them is illustrated. 

**Figure 7:**
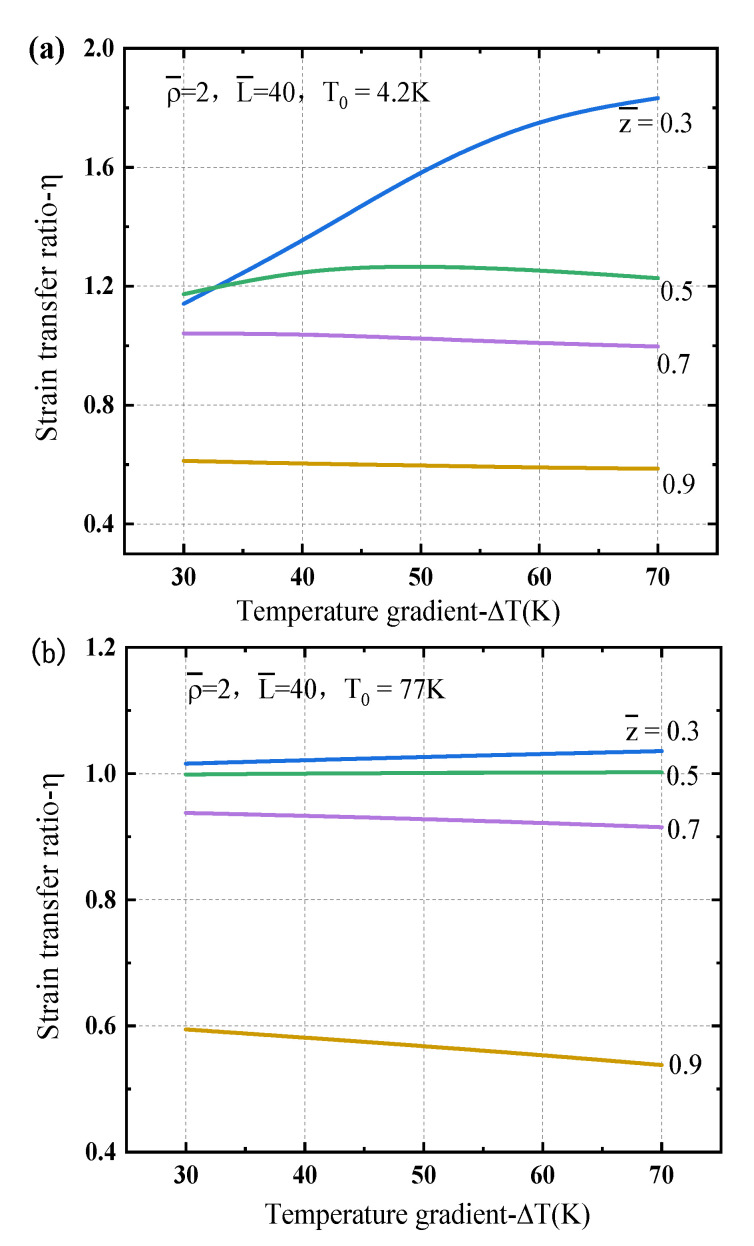
Strain transfer ratio of the optical fiber sensor dependence of the temperature gradient at different locations under a linear gradient temperature load: (**a**) $T_{0}=4.2\;K$, (**b**) $T_{0}=77\;K$.

Figure 7 plots the strain transfer ratio dependence of the temperature gradient at different positions along the sensor length to exhibit different characteristics with ΔT. One can see that the transfer ratios η are even larger than 1.0 near the left end while they are usually less than 1.0 near the right end of the sensor. Such feature is more obvious for liquid helium temperature region as shown in Figure 7a than that for liquid nitrogen temperature region as shown in Figure 7b. It is mainly because the material properties such as the difference of elastic modulus of the protective and matric layers, especially the CTEs of the protective and matrix layers at low temperature, as illustrated in Figure 3, which further results in discrepancies of strain states in the different layers. Even the strain in the protective layer is little higher than that in the matrix so that η is greater than 1.0 near the left end of the sensor structure. Such feature is more remarkable for a higher gradient temperature. 

**Figure 8:**
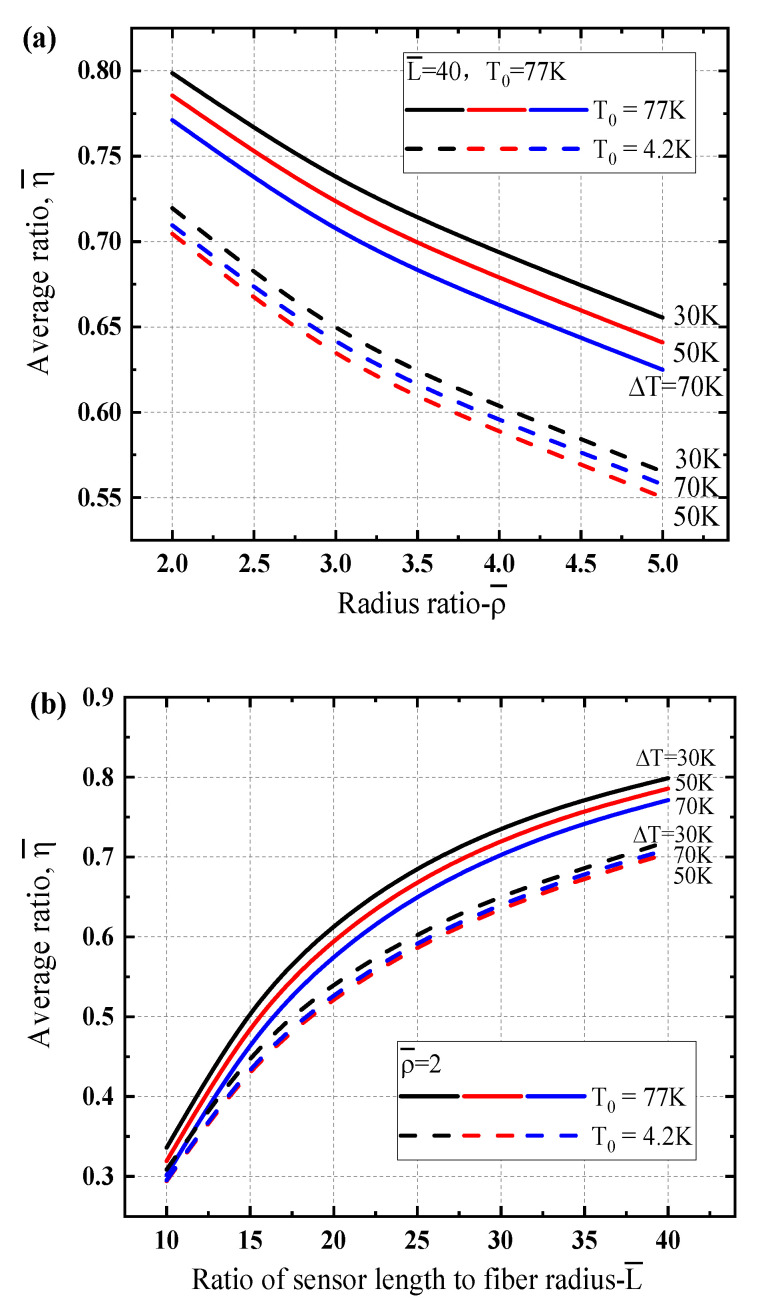
Comparison of average strain transfer ratio of the optical fiber sensor for different cryogenic temperature regions dependence of geometrical parameters under a linear gradient temperature load: (**a**) the radius ratio $\bar{\rho }$, (**b**) the ratio of sensor length to fiber radius $\bar{L}$.

To give a better understating on the strain transfer ratio of the sensing structure and their performance under different low temperature zones, the average ratios depending upon the geometrical parameters are presented in Figure 8. One can see that the average values are always less than 1.0 which is reasonable for the strain transfer mechanism of multi-layer structure. Figure 8a illustrates the average strain transfer ratio varying with the radius ratio of the protective layer thickness of the sensing structure. It can be found that the average transfer ratio decreases with the thickness of the protective layer and the values for liquid nitrogen region (i.e., $T_{0}=77\;K$) are larger than the ones for liquid helium region (i.e., $T_{0}=4.2\;K$). With the increase of the sensor length, the average strain transfer ratio increases obviously for different temperature gradients, as shown in Figure 8b. Reducing the thickness of the protective layer and increasing the sensor length can improve the strain transfer efficiency of the sensing structure at low temperature.

(b) Gaussian Temperature Distribution Load

In an extreme condition, a point-like heat source will be generated locally in the structure. The temperature and thermoelastic deformation near the hot spot approximately distribute according to a bell curve. For example, a recent investigation of a spatially distributed fiber-optic sensor designed for temperature measurements in the steel industry was attempted, where a high temperature was generated by small point-like heating elements [37]. We consider the sensing structure is affected by a Gaussian temperature distribution in the form of $T_{1}(\bar{z})=T_{0}+f(\bar{z})\delta T$, where $f(\bar{z})=e^{A\cdot {\left(\bar{z}-0.5\right)}^{2}}$ represents the Gaussian distribution function and A=−4ln2 with a half-height width of 1, the temperature peak located at the midpoint ($\bar{z}=0.5$). The strain in the matrix is evaluated by $\varepsilon_{M}(\bar{z})=\int_{T_{0}}^{T_{1}(\bar{z})}\alpha_{M}(T)dT$, and the different low temperature regions of liquid helium and nitrogen are considered (e.g., $T_{0}=4.2\;K,77\;K$).

**Figure 9:**
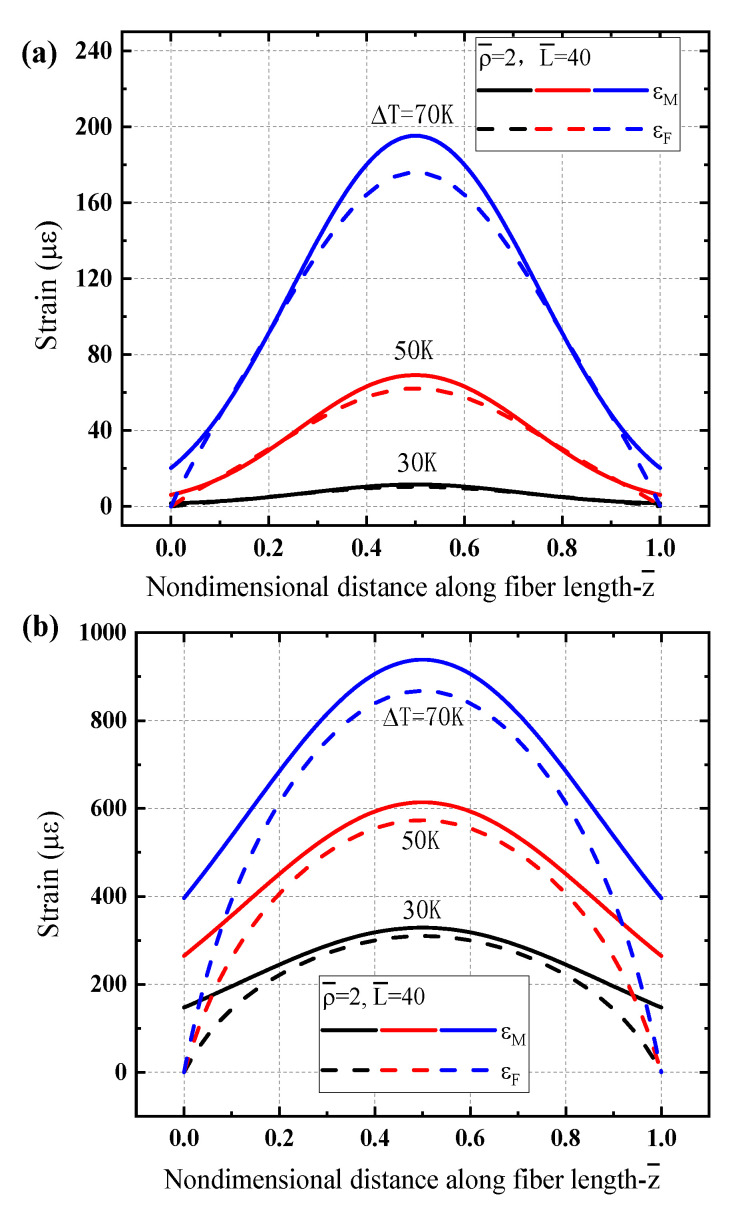
Strain characteristic of the optical fiber sensor under a Gaussian temperature gradient load: (**a**) $T_{0}=4.2\;K$; (**b**) $T_{0}=77\;K$.

Figure 9 illustrates the strain distributions in the matrix and the fiber core. One can see that the strain response of the optical fiber core is not able to reflect completely the true matrix strain, and the thermoelastic strain of the matrix caused by the temperature rise at the middle position is usually higher than the evaluated one of the optical fiber core, while the feature is reverse near the two ends of the sensor part. For a larger temperature peak, it shows a significant discrepancy of strains in the matrix and fiber core. There exhibit the similar features for different low temperature regions (as shown in Figure 9a,b). These discrepancy of strain distributions in the different layers is mainly caused by the temperature-dependent material properties of the layers particularly at different low temperature zones. The deformation configuration generated by the point heat source is complicated, and our model gives a possible method to explain and correct this inconsistent error. However, due to the errors introduced by the temperature and strain gradient, the difference of the real strain values measured and the predictions at each position is required further analysis.

**Figure 10:**
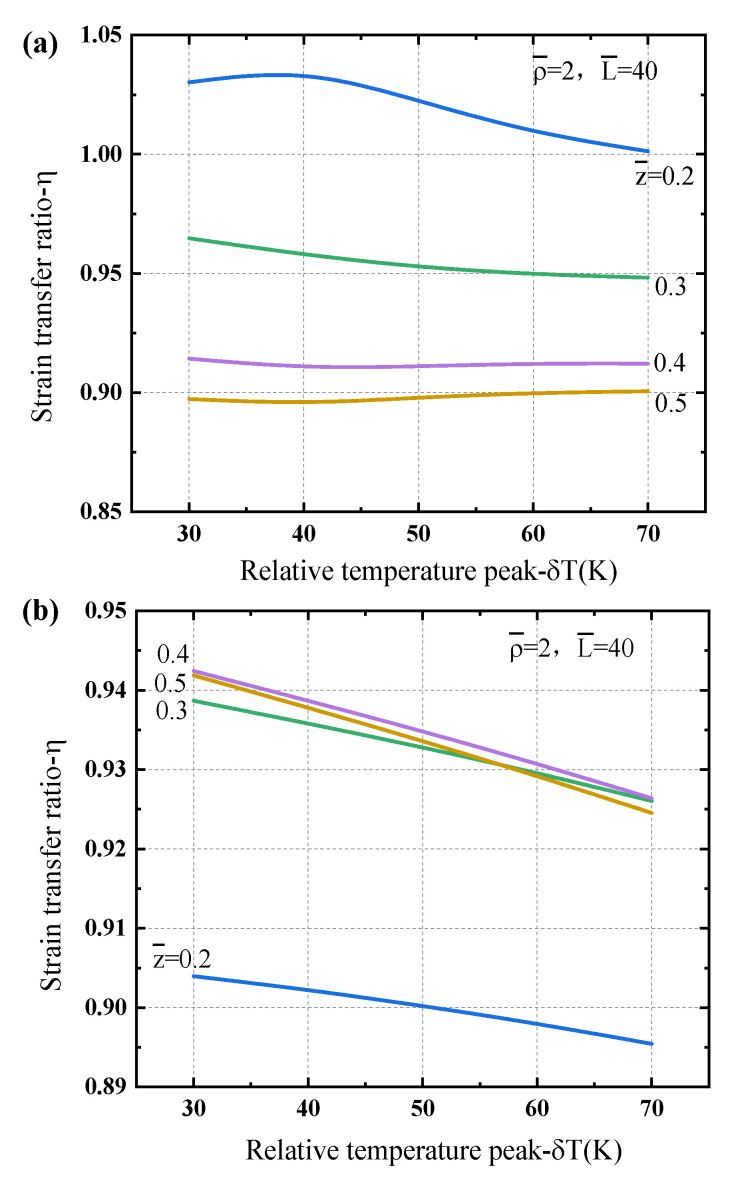
Strain transfer ratio of the optical fiber sensor dependence of the temperature peak at different locations under a Gaussian temperature gradient load: (**a**) $T_{0}=4.2\;K$, (**b**) $T_{0}=77\;K$.

**Figure 11:**
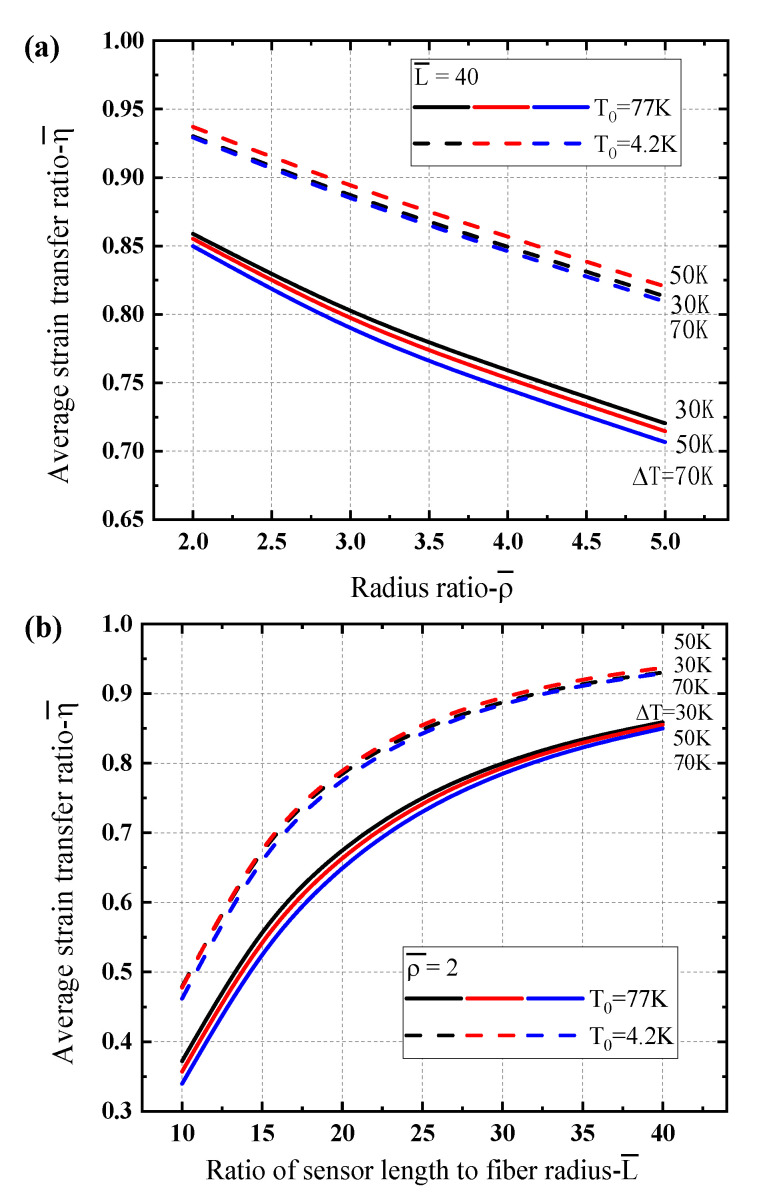
Comparison of average strain transfer ratio of the optical fiber sensor for different cryogenic temperature regions dependence of geometrical parameters under a Gaussian temperature gradient load: (**a**) the radius ratio $\bar{\rho }$, (**b**) the ratio of sensor length to fiber radius $\bar{L}$.

Figure 10 shows the strain transfer ratio dependence of the temperature peak for the Gaussian temperature gradient case at different positions along the sensor length to exhibit different characteristics. At the different positions along the sensor, there are quite different strain transfer ratios which are slightly larger than or less than 1.0, it has the similar mechanism as presented in the case of a linear gradient temperature load. The average transfer ratios depending upon the geometrical parameters are further presented in Figure 11. It clearly shows that the average strain transfer ratios decrease linearly as the thickness of the protective layer increases, while they increase nonlinearly as the sensor length. The average ratios are usually higher for the sensing structure under the liquid helium temperature region (e.g., $T_{0}=4.2\;K$) that that under the liquid nitrogen temperature region (e.g., $T_{0}=77\;K$). We also noticed that the influence of temperature peak on the average strain transfer ratio is not obvious. Based on the global performance of the optical fiber sensing structure, the protective layer always plays a main role for the strain transfer, the optimization geometrical parameters should be carefully designed.

## 4. Experiment Investigation at Low Temperature

In order to verify the theory that the strain transfer characteristic of the optical fiber sensing structure is related to temperature, strain measurement of a distributed optical fiber sensor at low temperature has been examined. An OFDR based on Rayleigh backscattering in the single-mode optical fiber was used to analyze the signals. The Rayleigh backscattering spectra (RBS) shift is affected by refractive index, which is determined by temperature and strain. The RBS shift Δλ of optical fiber bonded to a structure caused only by temperature ΔT can be expressed as follow,

(21) $$\frac{\Delta \lambda }{\lambda }=\xi \Delta T+\left(1-P_{e}\right)\alpha \Delta T$$

where α is the thermal expansion coefficient of the structure material, ξ is the thermo-optic coefficient of the optical fiber material, $P_{e}$ is the elasto-optic coefficient of the optical fiber material, and λ is the RBS.

LUNA ODiSI 6100 optical distributed sensor interrogator was utilized to record the RBS shift of the optical fiber. A 0.65-mm spatial resolution can be obtained when the sampling rate is 20 Hz. The temperature data acquisition (DAQ) system based on the LabVIEW software and NI devices is developed to measure the temperature distributions of the samples.

### 4.1. Uniform Temperature Change

**Figure 12:**
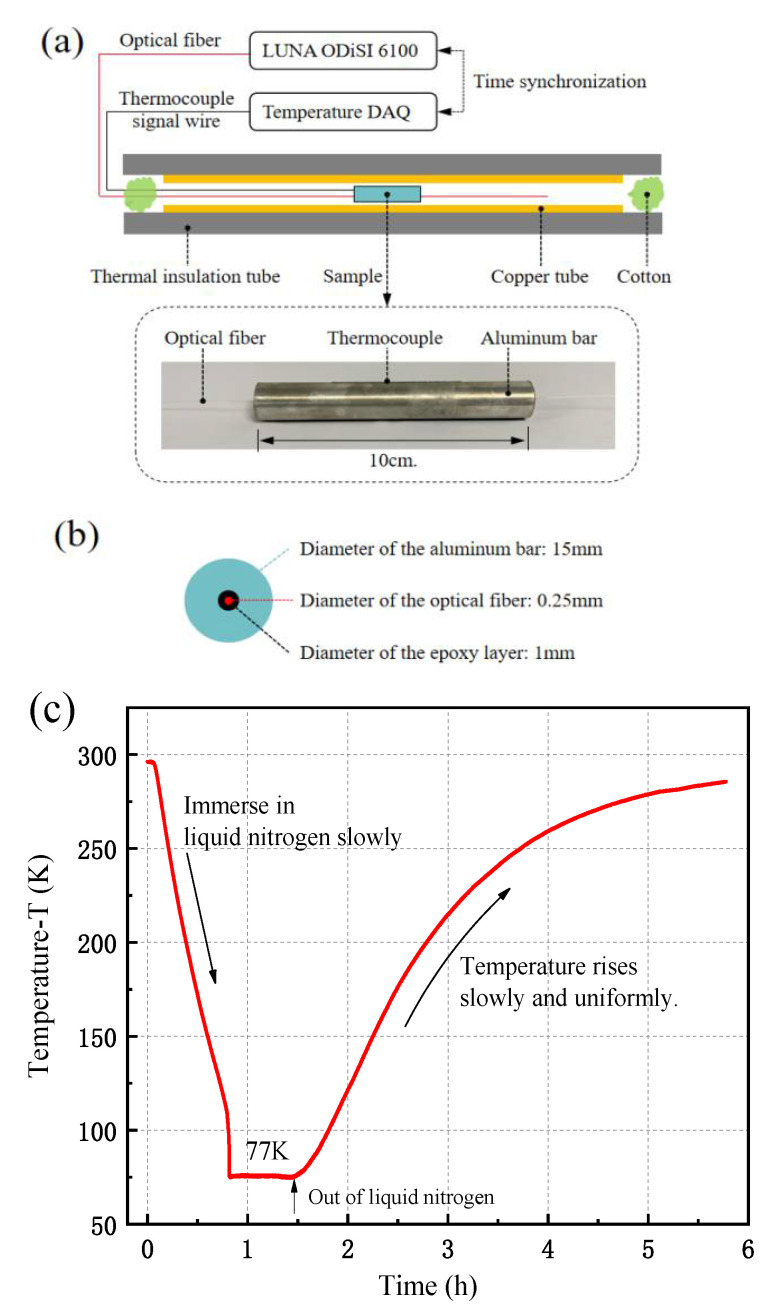
Optical fiber sensor embedded in an aluminum bar under uniform temperature load: (**a**) schematic diagram of experiment device and DAQ system, (**b**) cross section of the sample, (**c**) temperature variation of the sample measured by thermocouples.

The thermal strain related to RBS shift signal of an optical fiber for a uniform temperature variation from 77 K to 289 K has been measured. To obtain a uniform temperature load, a sample with the optical fiber sensor is placed in a thick copper tube coated with thermal insulation material, and cotton is filled at both ends of the copper tube to prevent convection with the ambient air, as shown in Figure 12a. The optical fiber RBS shift signal is collected by LUNA ODiSI 6100 interrogator which is time synchronized with the temperature acquisition device, and the RBS shift is directly associated with temperature. The sample is a T6061 aluminum bar embedded with Corning SMF-28 Ultra optical fiber. The bonding material is STYCAST 2850 FT epoxy resin, and its geometrical dimensions are shown in Figure 12b. Since the bonding layer thickness is quite thicker than the fiber core it can be taken as one layer of the multi-layer structure. The initial low-temperature of 77 K can be obtained by immersing the sample in liquid nitrogen. With the liquid nitrogen removed, temperature of the sample returns to the room temperate (e.g., 289 K) very slowly and uniformly by the natural recovery, as shown in Figure 12c.

**Figure 13:**
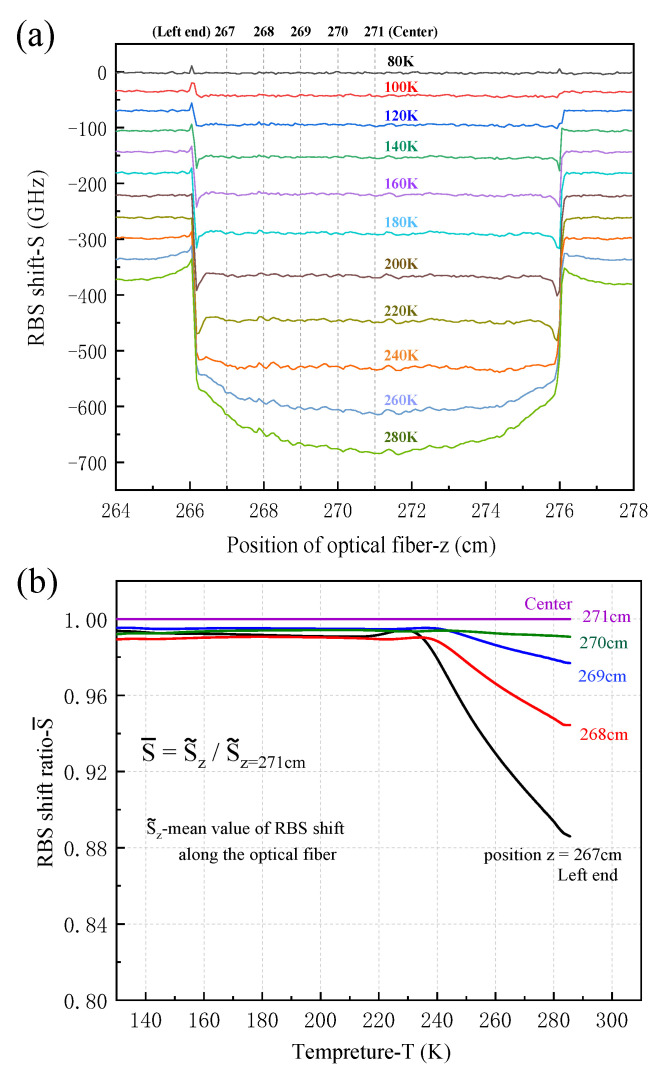
RBS shifts of the sample embedded with optical fiber sensor: (**a**) RBS shift of optical fiber at different temperatures, (**b**) RBS shift ratio at different positions along the sample.

Taking the RBS shift (S) at 77 K as the reference value, the distribution of RBS shift in the sample for different position (z) and temperatures (T) is presented in Figure 13a. Good RBS shift signals were obtained except for the abnormal signals near two ends (e.g., z = 266 cm and 276 cm) of the sample. Small fluctuations at some locations are likely due to minor defects in the epoxy resin or at the boundary condition, but do not affect the overall measurement. During temperature rising slowly, the temperature distribution of the aluminum bar is uniform and stable, and its thermal deformation is therefore uniform. The influence of thermo-optic effect on the optical fiber RBS shift is the same. At the same temperature, the difference of RBS shift along the fiber is only contributed from the deformation measured by the optical fiber. At very low temperature range from 77 K to 220 K, the RBS shifts at different positions along the sample have almost the same value except at the two ends. When the temperature is up to 260 K and a higher value, the RBS shift diagram is consistent with the prediction of the theoretical model. The measured values in the middle region of the sample are large and those near two ends are small. It indicates that the strain transfer ratio near the ends decreases and a higher value is obtained at the midpoint of the sample. The main reason is that the epoxy resin has a large elastic modulus at low temperature, so that the high strain transfer ratio is obtained. With the increase of temperature, the epoxy resin becomes soft, the elastic modulus decreases, and the strain transfer ratio becomes low.

In Figure 13b, the RBS shift ratio ($\bar{S}$) of the sample at different temperatures is compared. In order to reduce the fluctuation caused by nonuniformity, the average value of RBS shift ($\tilde{S}$) within 0.5 cm length around a certain position z_0_ of optical fiber (z_0_ − 0.5 < z < z_0_ + 0.5) is introduced. When the temperature is less than 220 K, the RBS shift ratio of each point is around 0.99. When the temperature is higher than 240 K, the RBS shift ratio decreases obviously, and the near to the end, the decrease is obvious. The experimental results show that low temperature is beneficial to strain transfer to some extent.

### 4.2. Temperature with Great Gradient

**Figure 14:**
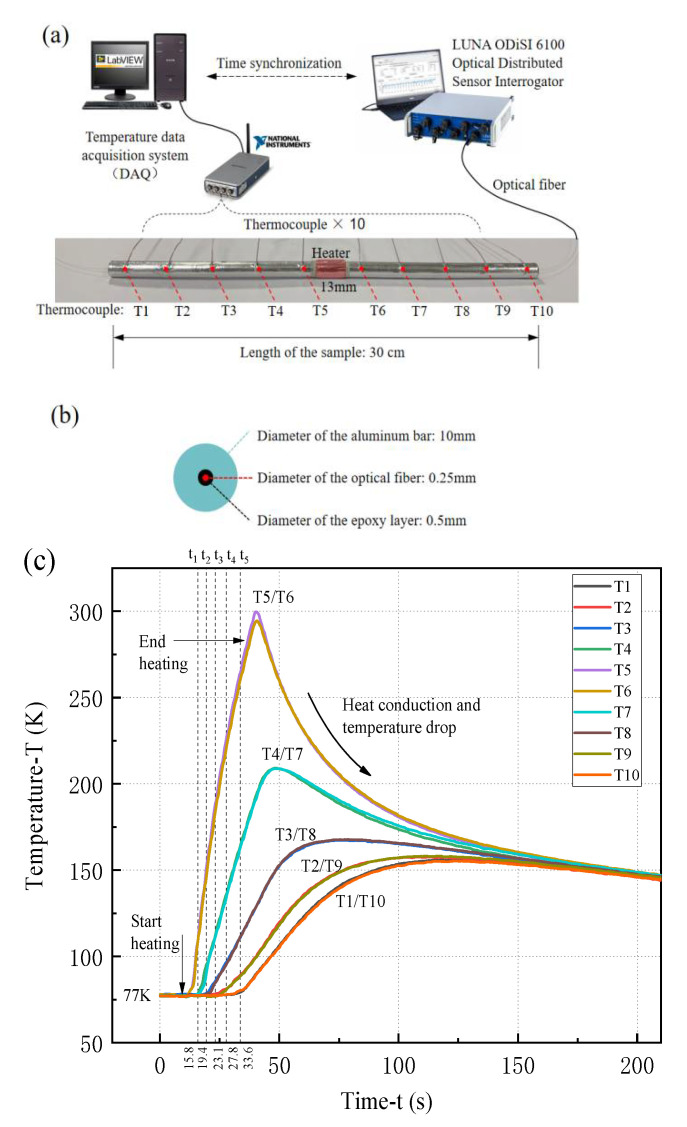
Aluminum bar embedded with optical fiber under gradient temperature load: (**a**) schematic diagram of the experiment instrument and sample, (**b**) cross section of the sample, (**c**) temperature characteristic of the sample measured by thermocouples.

We further make a measurement on the thermal strain of a sample with optical fiber under a temperature gradient load. As shown in Figure 14a, a winding resistance heater of power supply of 100 W is set at the midpoint of an aluminum bar sample with length of 30 cm. There are 10 tiny thermocouples (named as T1, T2, …, T10) evenly arranged along the sample to measure the rapidly changing temperature, whose response frequency is above 10 Hz. The data acquisition method is the same as the previous experiment. In order to obtain accurate measurement, the epoxy resin diameter in the sample is adjusted to 0.5 mm, and the diameter of aluminum rod is reduced to 10 mm for better temperature conduction, as shown in Figure 14b. The sample was cooled to 77 K with liquid nitrogen, and then removed from the liquid nitrogen and supplied power to the heater at the same time. When the thermocouples T5 or T6 reaches 300 K, we turn off the heater, and the heat conduction will make the temperature redistributed evenly, as shown in Figure 14c. The temperatures at the symmetrical positions measured by the thermocouples are very close, such as T5/T6, T4/T7, T3/T8, T2/T9, and T1/T10. During the heating period, the RBS shifts at different times (e.g., t_1_, t_2_, t_3_, t_4_, and t_5_) are extracted for the subsequent analysis.

**Figure 15:**
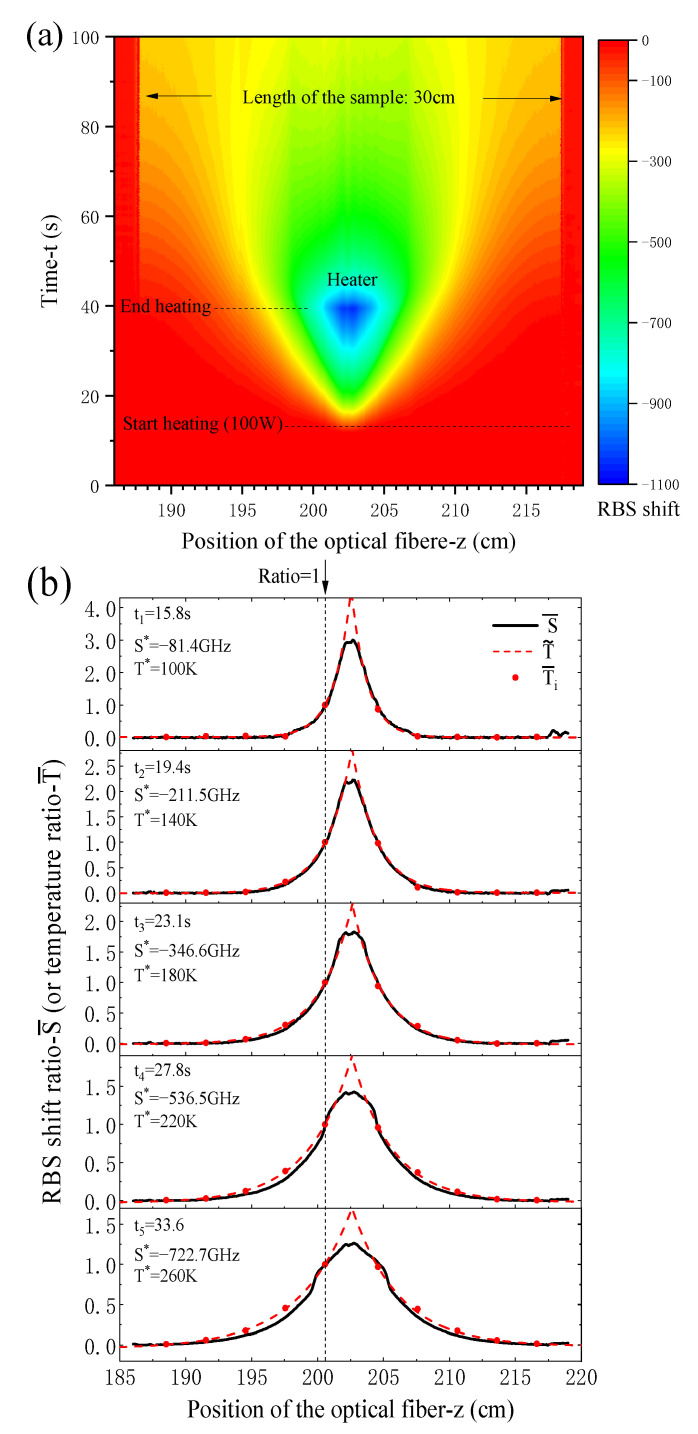
RBS shift map of the sample measured by optical fiber sensor: (**a**) 2D evolution of RBS shift with time and position, (**b**) RBS shift ratio (or temperature ratio) at different position along the optical fiber.

The evolution of RBS shift along the optical fiber sensor with time is shown in Figure 15a. When the heater is triggered to work (at t = 13 s), which results in a temperature rise at the middle area of the sample, and the heat then propagates in the bar. In the initial stage of heating, the heat does not propagate to the ends and the temperature in most area of the sample still keeps 77 K, so the RBS shift remains zero, as shown the red color in the figure. The optical fibers, outside the sample (z < 188 and z > 218), are always unaffected by the heater, so RBS shift is also zero. The heater is turned off when the value of T5/T6 reaches 300 K (at t = 40 s) in the experiment. As seen from the figure, the RBS shift values near the heater area are high, and the heat is transferred quickly, and the temperature is gradually changed during the heating and heat conduction processes.

For comparation, the RBS shift and temperature at five times (t1, t2, t3, t4, and t5) are presented in Figure 15b, in which the dimensionless values of RBS shift ratio $\bar{S}=S/S^{*}$ and temperature ratio $\bar{T}=\left(T-77\right)/\left(T^{*}-77\right)$ respectively are used (the reference values of RBS shift $S^{*}$ and temperature $T^{*}$ are chosen at z = 200.5 cm). From the figure, one can see that the continuous distribution curves of RBS shift ratios detected by the optical fiber sensor have good consistency with the temperatures ratio at 10 points measured by thermocouples. Furthermore, to obtain the temperature distribution along the sample a fitting function $\tilde{T}=A_{1}+A_{2}\exp(A_{3}z^{2}+A_{4}z+A_{5})$ is given by the measured temperatures, where $A_{i}\left(i=1,2,3,4,5\right)$ denote the fitting parameters. One can see that the fitting function of the real temperature distribution approximatively correspond to an analogous Gaussian temperature distribution load. From Figure 15b, one also can find that at the five times the ten temperature ratios match the RBS shift very well, and good consistency is obtained in all parts of the sample except for the resistance heater region. The main characteristics of the RBS shift ratios have reasonable comparability with the thermal strains qualitatively as predicted in the previous section for a Gaussian temperature distribution in the sensing structure. Additionally, high temperature which means low elastic modulus of epoxy and great gradient, make the strain transfer ratio smaller around the heater as the theoretical predictions, so that the strain measured by optical fiber sensor has a big difference with the true value under this condition. However, in the most area far away from the heater there always present good measurements, and the values near the heater can be deduced by proper fitting function of RBS shifts in the practice.

## 5. Conclusions

The strain transfer characteristic of a three-layer sensing model based on strain optical fiber sensors at low temperature has been studied theoretically and experimentally. Since the harsh working conditions for the sensing structure, the Young’s modulus and CTE of the materials both are temperature-dependent. The different thermal loads including a constant temperature variation and temperature gradients have been considered for the optical sensing structure. The following conclusions can be drawn from the investigation:

(1) The proposed sensing model can successfully capture the strain transfer characteristics of the three-layer optical sensor structure as a temperature gradient exists, and the deformation in the different layers were accurately obtained. Meanwhile, a traditional model under uniform temperature loading for strain transfer analysis has been gained by the proposed model as a degradation form.
(2) With temperature decreasing, the Young’s modulus of the protective layer of the optical sensor always increases so that a quite good strain transfer performance is achieved. It results in the measurement of optical fiber strain sensor being more reliable and accurate under low temperature than that at room temperature.
(3) Since the temperature-dependent properties of layers of the fiber sensor, the strain transfer ratios are even larger than 1.0 near the sensor ends at low temperature and a high gradient temperature load, while the average strain transfer ratios are commonly less than 1.0. The protective layer always plays a main role for the strain transfer for the global performance of the optical fiber sensing structure, and the optimization geometrical parameters should be carefully designed which can be improved by reducing thickness of the protective layer and increasing sensor length of the multi-layer sensing structure.
(4) The experiments on a sample embedded with an optical fiber sensor were conducted. The thermal strains related to RBS shifts of the optical fiber for a uniform temperature variation and a temperature gradient load heated by a resistance heater were measured, to qualitatively verify the theoretical predictions on the main characteristics under low temperature condition.

## Data Availability

Not applicable.
